# ISCB Honors David Haussler and Aviv Regev

**DOI:** 10.1371/journal.pcbi.1000101

**Published:** 2008-07-18

**Authors:** Clare Sansom, B. J. Morrison McKay

**Affiliations:** 1 Birkbeck College, London, United Kingdom; 2 International Society for Computational Biology (ISCB), University of California San Diego, La Jolla, California, United States of America; University of California San Diego, United States of America

Each year, the International Society for Computational Biology (ISCB; http://www.iscb.org) gives two major awards to leaders and innovators in the field of bioinformatics. The awards committee, composed of current and past directors of the Society and previous award winners, has announced that the 2008 Accomplishment by a Senior Scientist Award will be given to David Haussler of the University of California Santa Cruz, Santa Cruz, California, United States, and the 2008 Overton Prize for outstanding achievement in early to midde career will be awarded to Aviv Regev of MIT and the Broad Institute of MIT and Harvard, Cambridge, Massachusetts, United States. Both award winners are Associate Editors for *PLoS Computational Biology*, an official journal of ISCB.

Søren Brunak, director of the Center for Biological Sequence Analysis at Technical University of Denmark and ISCB Awards Committee chair, admitted that the choice of award winners had not been easy. “These awards are a sign of recognition of achievement not just from ISCB, but from the whole bioinformatics community,” he said. “It is a significant honor to receive one.” He highlighted the fact that the 2008 award winners had made major contributions to both the development of algorithms and the application of those algorithms to “real life” biological problems.

Both awards will be presented at the Society's flagship international conference, ISMB (Intelligent Systems for Molecular Biology) 2008, where the winners will give *Keynote Presentations*. The Sixteenth Annual ISMB Conference will take place July 19–23 in Toronto, Canada.

## ISCB 2008 Accomplishment by a Senior Scientist Award: David Haussler

**Image 1 pcbi-1000101-g001:**
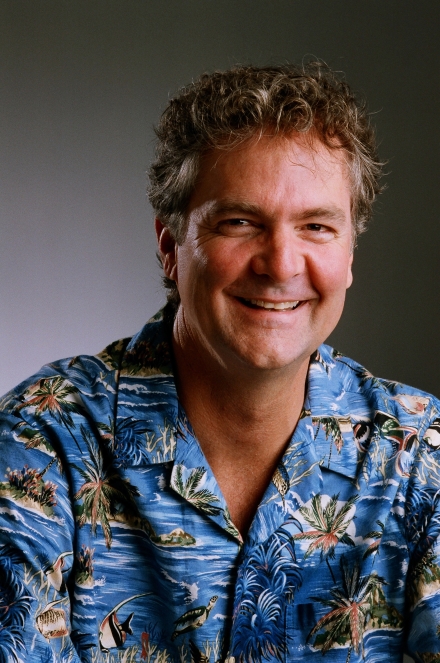
David Haussler

Bioinformaticians worldwide owe a debt to David Haussler (see Image 1) for his significant contributions to the design of algorithms they use every day. In this, he is like his two immediate predecessors as Senior Scientist Accomplishment Award winners, Temple D. Smith and Michael S. Waterman, the eponymous joint authors of the Smith–Waterman algorithm for local alignment of DNA or protein sequence fragments. “Haussler's group was one of the pioneers of machine learning in bioinformatics, introducing Hidden Markov Models for the statistical analysis of patterns in biological data,” says Brunak. However, Haussler's recent achievements have been more in the application of bioinformatics methods than in their development. Since 1999, he has been one of the principal figures in sequencing, and later analysing, the human genome and those of other mammals, and in mining this genomic information for insight into vertebrate evolutionary history.

Haussler originally trained as a mathematician, graduating magna cum laude from Connecticut College and obtaining prizes for mathematics at both Bachelor's and Master's levels. His first encounter with computational biology came in graduate school, at the University of Boulder in Colorado, where he had the good fortune to study for his Ph.D. under Andrzej Ehrenfeucht. “Andrzej is an extraordinary man,” says Haussler. “In our weekly research seminars, we would discuss topics ranging from dinosaur flight to abstract graph theory. He taught me that I should never be constrained by disciplinary boundaries, and never be frightened to tackle big problems. The word “bioinformatics” didn't exist when I was a graduate student, but we were doing it.” Two of his fellow students, Gary Stormo and Gene Myers, have also gone on to have distinguished careers in the field. Stormo, now professor of genetics at the University of Washington in St. Louis, and Deputy Editor-in-Chief of *PLoS Computational Biology*, has made significant contributions to the study of DNA–protein interactions and the prediction of nucleic acid structure and function; Myers was one of the inventors of the BLAST program, a key innovator in shotgun sequencing, and a principal architect of Celera's draft sequence of the human genome.

Haussler's first years as an independent investigator were devoted to rather abstruse studies in pattern recognition and machine learning, focusing on modelling the way the brain learns. He only shifted from computational neuroscience back to bioinformatics when Anders Krogh joined him at Santa Cruz as a post-doc. Characteristically, Haussler underestimates his own role in their joint achievements. “Anders was an exceptional post-doc, who has gone on to have an exceptional career as an independent scientist. He came to my lab to work on machine learning, but soon discovered that these methods could be applied to biological sequence analysis, to classifying proteins into families and recognising genes in fragments of DNA.” Krogh is co-author of acclaimed and popular textbook *Biological Sequence Analysis: Probabilistic Models of Proteins and Nucleic Acids*. Other members of Haussler's group applied machine learning techniques to the classification of microarray data, including the development of one of the first expression-based methods for distinguishing tumor from normal cells.

Late in 1999, a phone call changed the direction of Haussler's research. “I was called by Eric Lander, one of the leaders of the public human genome sequencing project, and asked to apply my HMM methodology to identifying the genes in the then newly sequenced human DNA,” he explains. At that time, the public project was in a “full-on race” with Celera to publish an initial working draft of the sequence. Haussler joined the international public effort, rapidly recruiting a team of talented young bioinformaticians that included Jim Kent, winner of ISCB's 2003 Overton Prize.

Barely six months after Haussler joined the project, both teams—the publicly funded one and Celera's—were ready to release their first genome drafts into the public domain. Haussler well recalls July 7, 2000, when the complete draft genome sequence was posted on the University of Santa Cruz' Web server. “Seeing the waterfall of As, Gs, Cs, and Ts pouring off our server was an emotional moment,” he says. “We were witnessing the product of more than three billion years of evolution, sequences passed down from the beginning of life to present-day humans.” This excitement was shared by the worldwide scientific community; Internet traffic on the Santa Cruz server reached 0.5 terabytes per day then: a record that still stands.

Raw DNA sequence, however, is not much use on its own, and Haussler has dedicated the first years of the new millennium to mapping and analysing that sequence. The first release of Santa Cruz' genome browser went online shortly after the human sequence was released, and it now includes twenty complete vertebrate genome sequences, plus those of a few representative invertebrates. “The publication of the second vertebrate genome—that of the mouse—gave us the first real sequence-based insights into the mechanisms of vertebrate evolution,” he says. “And we could also use evolutionary theory and sequence analysis to answer a central question: how much of the mammalian genome is ‘junk’?” Assuming that fewer inter-species substitutions are found in functional DNA than in non-functional DNA, Haussler's team in the mouse genomics consortium were able to estimate that at least 5% of a mammalian genome is functionally important. This value has been confirmed as more complete sequences have emerged. “We may think that 5% is a small value, but it is particularly interesting in that less than 1.5% of the genome codes for proteins. There is still a question over the function of much of the 3.5% that is conserved but does not form protein-coding genes.” Other questions that have attracted Haussler's attention include the analysis of hyper-conserved DNA sequences that remain virtually unchanged in divergent species, and the genetic changes that distinguish humans from apes. While most researchers in this field have concentrated on gene gain during evolution, Haussler and his team recently identified twenty-six genes that are well-established in the vertebrate lineage but that were lost in the latter stages of human evolution.

## ISCB 2008 Overton Prize: Aviv Regev

**Image 2 pcbi-1000101-g002:**
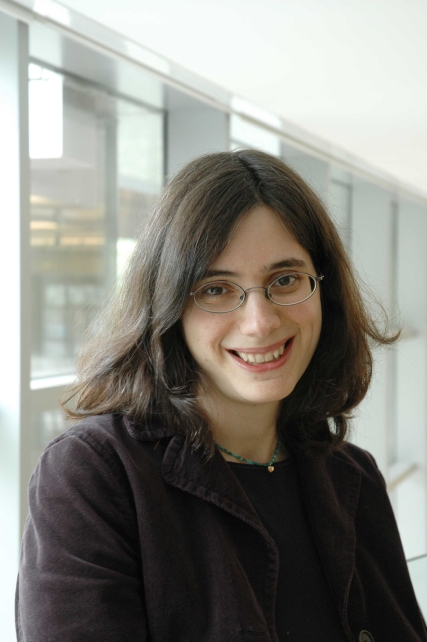
Aviv Regev

Brunak describes the 2008 Overton Prize winner Aviv Regev (see Image 2) as “a role model for how theoretical computer science can be applied to understanding biological organisms as systems.” Like Haussler, she has worked on both the development of computational methods and their applications, and she has already made significant contributions to both fields. Trained initially at Tel Aviv University, Tel Aviv, Israel, she entered an interdisciplinary undergraduate program but knew that her interests lay in bioinformatics “from Day One.” And she made her first contribution to the field while still an undergraduate, developing mathematical models for the evolution of DNA methylation. It was at that early stage that she realised the value of synergy between computational and “wet lab” biology. “There was no data for one critical phylogenetic group that I was studying, so I went to work in the lab at The Hebrew University to fill in the gaps,” she said. “This experience gave me a good idea of what lab work is like, and how important it is to anchor theoretical biology in to the real world.”

The idea that led directly to her graduate studies, however, came from a branch of computational science that at first glance has little, if any, connection with biology: pi calculus, typically applied to problems in electronic engineering. “I was listening to a conference talk by computer scientist Robin Milner, on the application of pi calculus to dynamic communication networks, when it occurred to me that molecular networks can have similar properties,” she explains. Following this up, she developed a method for describing and understanding the dynamic relationships between entities in a biological system (such as proteins in an interaction network) using this type of “process algebra.” “Regev started out as a young graduate student applying a novel and rather obscure computational methodology to a biological problem. This type of work has only very recently been recognised as a major part of systems biology,” says Brunak. The Overton Prize is awarded in memory of former ISCB director Chris Overton, who died prematurely in 2000; his research output was always innovative and thought-provoking, and it seems particularly fitting that this prize should go to a young researcher whose graduate studies were characterised by such an unexpected—and productive—interdisciplinary leap.

After graduation, Regev moved to the US to take up her first independent position, at the Bauer Center for Genomics Research at Harvard University. “As a Bauer Fellow, I had five years' guaranteed money to start my own group, with no teaching or admin responsibilities,” she says. “It was research heaven.” There, her research interests switched to the use of probabilistic graphical models to reconstruct networks based on genomic and transcription data, using yeast as a model system. Her group, in collaboration with Amos Tanay and Ron Shamir, showed that while some DNA sequences involved in gene regulation are tightly conserved across even distantly related yeast species, other such sequences diverge, and used this to infer the evolutionary history of certain regulatory elements in yeast. “Our work was vindicated when a paper from an experimental group in *Molecular Cell* showed the transitional event that we had predicted,” she says. “That was great motivation!”

In 2006, Regev took up a position as an assistant professor at MIT, where she is also a Core Member of the Broad Institute. She has extended her network models to a range of applications including the characterisation of genes that are co-expressed in a range of cancer types but not in normal cells, and studying gene duplication. And, once again, a chance meeting sparked a productive idea. “I was returning from a conference with a colleague, Jill Mesirov, who had been trying to study variation in the gene expression of the malaria parasite in different patients' blood cells,” she explains. “Mesirov's data came from Johanna Daily and Dyann Wirth, infectious disease specialists from Harvard, who suspected that variation in gene expression might explain some of the observed variation in the clinical course of the disease. I wondered whether there might be equivalence to my own classification of yeast gene expression patterns, and so it proved: the malaria samples could be classified into three groups, similar to states characteristic of active growth, a starvation response, and a stress response in yeast.” This work was published in *Nature* in December 2007, where it was also featured in that journal's *Making the Paper* section.

This is not the first time that Regev's work has been recognised by the ISCB. During the last decade, her name has appeared on a prize-winning poster or paper abstract at ISMB no fewer than four times. The Overton award is the most prestigious of her career so far, but it is unlikely to be the last.

## Additional Information

For the full agenda and registration information for ISMB 2008, where these ISCB award winners will be joined by six other distinguished *Keynote* lecturers, and which will also feature a *Highlights Track*, *Special Sessions*, *Technical Demonstrations*, and a unique “Visual Reflections on Science” exhibition, please visit the conference Web site at http://www.iscb.org/ismbeccb2008.

For a review of past ISCB Accomplishment by a Senior Scientist Award and Overton Prize winners, please see http://www.iscb.org/ssaa.shtml and http://www.iscb.org/overton.shtml, respectively.[Fig pcbi-1000101-g001] [Fig pcbi-1000101-g002]

